# Editorial: Acupuncture for pain management

**DOI:** 10.3389/fnins.2023.1235111

**Published:** 2023-06-20

**Authors:** Jun Zhou, Yuke Teng, Xinyue Zhang, Shirui Cheng, Xinling Li, Shengjie Hu, Weiming Luo, Xingyao Chen, Zhengjie Li

**Affiliations:** ^1^Acupuncture and Tuina School, Chengdu University of Traditional Chinese Medicine, Chengdu, China; ^2^Acupuncture and Brain Research Center, Chengdu University of Traditional Chinese Medicine, Chengdu, China

**Keywords:** pain management, acupuncture, efficacy, mechanisms, evidence-based, clinical applications

Pain management poses significant challenges worldwide due to the high prevalence, debilitating effects, and limited effective medication options. In light of the opioid crisis and concerns about drug abuse, regulatory and oversight agencies are advocating for alternative treatment options for pain control. Acupuncture is a potential choice to address these demands. However, despite its growing popularity, there is still limited understanding of the scientific mechanisms underlying acupuncture's effectiveness for pain management, and there is a need for translational research to bridge the gap between mechanisms illustration and clinical applications. To address these gaps, we launched a Research Topic about “*Acupuncture for pain management”* on November 10th, 2021, inviting researchers to contribute their studies and identify potential avenues for future research in this field.

The Research Topic “*Acupuncture for pain management*,” published in *Frontiers in Neuroscience*, featured 19 articles involving 161 authors from 5 countries, presenting significant contributions to our understanding of acupuncture's efficacy, safety, and mechanisms for pain management. The articles can be summarized into four categories: efficacy of acupuncture, acupuncture for specific pain conditions, mechanisms illustration of acupuncture analgesia, and technological advances in acupuncture and personalized treatment integration.

(1) Efficacy of Acupuncture: Several studies demonstrated the effectiveness of acupuncture in various pain conditions, including migraines, neck pain, shoulder pain, and osteoarthritis. For instance, Liu C-T. et al. conducted a multicenter randomized controlled trial and found that acupuncture could effectively prevent menstruation-related migraines. Shi et al. conducted a randomized controlled trial evaluating the efficacy and safety of electro-thumbtack needle therapy for chronic neck pain. Heo et al. and Zhan et al. confirmed the benefits of acupuncture in systematic reviews and meta-analyses for shoulder pain in scapulohumeral periarthritis and post-stroke shoulder pain, respectively. Additionally, Hee et al. conducted an overview of systematic reviews and meta-analyses, showing the effectiveness of warm needle acupuncture in treating osteoarthritis. These studies provide evidences for the efficacy of acupuncture in various pain conditions.

(2) Acupuncture for Specific Pain Conditions: Several studies highlighted the efficacy of acupuncture in managing pain associated with traumatic rib fractures, irritable bowel syndrome, and acute renal colic. Liu L-Y. et al. demonstrated that acupuncture was a safe and effective analgesic modality for managing pain in traumatic rib fractures. Yang et al. conducted a systematic review and meta-analysis, finding that acupuncture and moxibustion were effective treatments for irritable bowel syndrome. Chen L. et al. conducted a prospective cohort study and found that acupressure provided significantly faster pain relief (for acute renal colic) compared to parecoxib sodium within 10 min. These studies provide evidences for the effectiveness of acupuncture in specific pain conditions.

(3) Mechanisms illustration of Acupuncture Analgesia: Several studies also provided insights into the mechanisms illustration underlying acupuncture's therapeutic effects. Acupuncture was found to modulate neuronal activity, pain-related ion channels, and the release of inflammatory cytokines and chemokines. It also activated the descending pain control system and demonstrated effects on brain regions involved in musculoskeletal pain and primary dysmenorrhea. Additionally, a proposed mechanism based on tensegrity principles offered potential for treating fibromyalgia. For instance, Ma et al. proposed that the somatosensory system played a potential role in the effectiveness of acupuncture for neuropathic pain. Acupuncture has the ability to inhibit neuronal activity induced by neuropathic pain through the reduction of pain-related ion channel activation and suppression of inflammatory cytokines and chemokines release. Furthermore, it could activate the descending pain control system by increasing the levels of spinal or cerebral neurotransmitters, such as 5-hydroxytryptamine (5HT), norepinephrine (NE), and opioid peptides. In another study, Wang J-y. et al. demonstrated that transcutaneous auricular vagus nerve stimulation (taVNS) increased plasma melatonin concentration, upregulated melatonin receptor (MTR) expression in the amygdala, and relieved peripheral neuropathic pain. Liu, Zhang et al. revealed a correlation between energy metabolism and acupuncture treatment of migraine through proteomic and metabolomic analyses. Moreover, Wang S. et al. discovered that electroacupuncture alleviated hyperalgesia in rats with acute neck pain by regulating neuronal-glial interactions and glutamate transporters. Ha et al. employed the coordinate-based activation likelihood estimation (ALE) method in a meta-analysis on acupuncture for musculoskeletal pain, revealing acupuncture-induced modulation of various brain regions in musculoskeletal pain patients. In the study by Liu, Li et al., acupuncture was found to modulate the functional connectivity density in patients with primary dysmenorrhea. Additionally, Plaut proposed a mechanism illustration for acupuncture as a global percutaneous needle fasciotomy based on tensegrity principles, providing insights into its potential for treating fibromyalgia. These findings shed light on the potential mechanisms illustration underlying acupuncture's efficacy in pain management and offer possible explanations for the observed clinical benefits of this therapeutic approach.

(4) Technological Advances in Acupuncture and Personalized Treatment Integration: Technological advancements in acupuncture and the integration of personalized treatment have expanded the capabilities of traditional acupuncture methods, facilitating customized treatments for individual patients. Fu et al. utilized innovative research strategies, such as machine learning and neuroimaging techniques, to identify potential biomarkers for discriminating patients with migraines and predicting the efficacy of transcutaneous vagus nerve stimulation (tVNS) in reducing migraine attack frequency. In a two-center randomized controlled trial, Chen C. et al. compared the CX-DZ-II smart electronic stimulator to the conventional SDZ-II electronic stimulator for alleviating neck pain caused by cervical spondylosis. The study demonstrated that the smart electronic stimulator was non-inferior to the conventional stimulator. Kim et al. published a protocol for an ongoing multicenter randomized placebo-controlled trial investigating the safety and efficacy of 650 nm invasive laser acupuncture for non-specific chronic low back pain. Furthermore, Li et al. plan to conduct a randomized controlled trial to investigate whether combining transcutaneous electrical acupoint stimulation with electroacupuncture can expedite postoperative recovery after abdominal surgery. These studies aim to facilitate more effective and targeted acupuncture clinical applications in pain management. These technological advancements in acupuncture and personalized treatment integration hold great promise for improving the effectiveness, precision, and accessibility of acupuncture therapy.

Acupuncture's therapeutic potential extends beyond its analgesic properties. Its holistic approach, focusing on the restoration of balance and harmony within the body, offers a unique perspective in the realm of pain management. With the world grappling with the ramifications of the opioid crisis and the limitations of satisfactory pharmacological options, it is imperative to explore alternative treatments that are safe, sustainable, and evidence-based. Acupuncture embodies these qualities and holds immense promise in transforming the landscape of pain management. Acupuncture offers several advantages as a pain management modality, including its non-pharmacological nature, individualized approach, and minimal side effects. However, certain limitations, such as the requirement for experienced acupuncturists, inter-individual variability, and the lack of standardized treatment protocols, should be acknowledged.

Based on the existing evidence, developing personalized treatment plans that incorporate acupuncture for pain management is crucial. Clinicians should consider the specific pain condition, patient characteristics, and clinical guidelines when determining the appropriateness of acupuncture. Additionally, exploring the integration of acupuncture with conventional medicine and other pain management strategies may offer comprehensive solutions for patients.

It is our belief that this Research Topic will serve as a catalyst for innovation and collaboration, bringing together experts from various disciplines to further our understanding of acupuncture's role in pain management. Through rigorous research and a multidimensional approach, we can harness the full potential of acupuncture to alleviate the suffering caused by pain. Together, we can pave the way for a paradigm shift in pain management, transforming it into a more sustainable and patient-centered endeavor.

## Author contributions

JZ: conceptualization, writing the original draft, and writing-review, and editing. YT, XZ, SC, XL, SH, WL, and XC: writing-review and editing. ZL: provided oversight and guidance as the corresponding author and contributed significantly to the editing and finalization of the manuscript. All authors approved the submitted version.

